# Engineering Bamboo Leaves Into 3D Macroporous Si@C Composites for Stable Lithium-Ion Battery Anodes

**DOI:** 10.3389/fchem.2022.882681

**Published:** 2022-04-07

**Authors:** Hao Wu, Yingying Jiang, Wenjun Liu, Hong Wen, Shihui Dong, Huan Chen, Liwei Su, Lianbang Wang

**Affiliations:** State Key Laboratory Breeding Base of Green Chemistry-Synthesis Technology, College of Chemical Engineering, Zhejiang University of Technology, Hangzhou, China

**Keywords:** silicon, bamboo leaves, magnesiothermic reduction, porous structure, anode materials, lithium-ion batteries

## Abstract

Silicon is considered as the most promising candidate for anodes of next generation lithium-ion batteries owing to its natural abundance and low Li-uptake potential. Building a macroporous structure would alleviate the volume variation and particle fracture of silicon anodes during cycling. However, the common approaches to fabricate macroporous silicon are complex, costly, and high energy-consuming. Herein, bamboo leaves are used as a sustainable and abundant resource to produce macroporous silicon via a scalable magnesiothermic reduction method. The obtained silicon inherits the natural interconnected network from the BLs and the mesopores from the BL-derived silica are engineered into macropores by selective etching after magnesiothermic reduction. These unique structural advantages lead to superior electrochemical performance with efficient electron/ion transport and cycling stability. The macroporous Si@C composite anodes deliver a high capacity of 1,247.7 mAh g^−1^ after 500 cycles at a current density of 1.0 A g^−1^ with a remarkable capacity retention of 98.8% and average Coulombic efficiency as high as 99.52% for the same cycle period. Furthermore, the rate capabilities of the Si@C composites are enhanced by conformal carbon coating, which enables the anode to deliver a capacity of 538.2 mAh g^−1^ at a high current density of 4.0 A g^−1^ after 1,000 deep cycles. Morphology characterization verifies the structural integrity of the macroporous Si@C composite anodes. This work demonstrated herein provides a simple, economical, and scalable route for the industrial production of macroporous Si anode materials utilizing BLs as a sustainable source for high-performance LIBs.

## Introduction

The demand for high-performance energy storage devices has substantially increased due to depletion of fossil fuels in the past few decades (Chu et al., 2017; Liu et al., 2018). Extensive interest has been focused on rechargeable lithium-ion batteries (LIBs) (Zhang et al., 2017; Sun et al., 2018) owing to their high energy density and long lifetime for diverse applications such as portable electronic devices and electric vehicles (Armand and Tarascon, 2008; Olivetti et al., 2017; Yi et al., 2019). Among numerous anode materials for next generation LIBs, silicon (Zhang et al., 2016; Son et al., 2017) is considered as the most promising candidate because of its natural abundance (Guan et al., 2018), low Li-uptake potential and high theoretical specific capacity (4,200 mAh g^−1^ for Li_22_Si_5_) (Krüner et al., 2018; Nzabahimana et al., 2018). However, two major issues impede the commercialization of silicon anodes. Specifically, the dramatic volume change (>300%) during Li alloying and dealloying processes (Jin et al., 2017) and poor electronic conductivity (Liang et al., 2014; Sun et al., 2022) of silicon result in drastic capacity decay (Xu et al., 2019) and inferior rate capabilities, respectively (Ozanam and Rosso, 2016). Therefore, two main strategies have been proposed to improve the electrochemical performance of the Si anodes. One is to design nanoscale structure of silicon (Luo et al., 2015; Xu et al., 2017) or incorporate pores (Zhang et al., 2016; Vrankovic et al., 2017) into silicon framework. Such structures (e.g., nanowires, nanotubes, hollow nanospheres and porous Si) (Chan et al., 2008; Yao et al., 2011; Wu et al., 2012; Zhang et al., 2014; Sun et al., 2021) can mitigate the strain and shorten the distance of the charge transport/ion diffusion. The other strategy is to combine silicon with other conductive/protective materials in an attempt to improve the electronic conductivity of the anode while simultaneously stabilizing the growth of solid electrolyte interface (SEI) on the surface of silicon (Hu et al., 2008; Terranova et al., 2014; Sun et al., 2022). On the basis of these two strategies, significant advances have been achieved in improving the lithium storage properties since intensive efforts were dedicated to the preparation of porous silicon composites. Nonetheless, critical challenges still lie ahead of large-scale application of porous silicon anodes in discovering a sustainable and abundant resource as well as developing a simple, low-cost and scalable synthesis procedure.

Since Sandhage et al. reported the conversion from diatoms into silicon by magnesiothermic reduction in 2007 (Bao et al., 2007), this method has been demonstrated as a facile and scalable route for the production of porous silicon. By controlling the reaction conditions on magnesiothermic reduction, the porous structure of the pristine silica can be retained in the resultant silicon (Cho et al., 2016; Entwistle et al., 2018). In recent years, silica-rich biomass materials (such as rice husks, reed leaves, sugarcane bagasse, bamboo charcoal, etc.) have been used as sustainable and abundant resources for the fabrication of porous silicon (Jung et al., 2013; Liu et al., 2015; Praneetha and Murugan, 2015; Zhang et al., 2018). However, the nanopores inherited from the natural structures of the biomass materials are insufficient to buffer the severe swelling of silicon during long-term cycling (Lin et al., 2015). Therefore, engineering silicon anodes with macropores that are more beneficial to the structural integrity and charge transport becomes vital to improve their cycling stability and rate capabilities. Bamboos, as typically fast-growing perennials, are distributed in tropical and subtropical to mild temperate regions with more than 14 million hectares worldwide (Wang et al., 2015). In contrast to its culms and roots, bamboo leaves (BLs) are rarely commercially utilized despite a huge amount of waste BLs generated annually. For instance, the waste BLs weigh about 10^7^ metric tons in China, India and Japan per year (Umemura and Takenaka, 2014). Nonetheless, it has been reported that silica accounts for ∼17.4–23.1 wt% of the dried BLs (Kow et al., 2016), depending on species, climate and growing areas (Ikegami et al., 2014). More importantly, silica in BLs has evolved a robust network with well-defined mesoporous structure (Zhang et al., 2018). These unique characteristics allow BLs to be a suitable source for high-value anode materials of LIBs.

In this study, we report the recycling of BLs to produce 3D macroporous silicon via magnesiothermic reduction. The natural interconnected network of silica in BLs was preserved in the as-prepared silicon, while the mesopores from pristine silica were enlarged to macropores after selective etching. A conformal carbon coating was further applied to the 3D macroporous silicon to enhance the rate capabilities and structural stability. Owing to the durability of the structure, the anodes delivered a high capacity of 1,247.7 mAh g^−1^ after 500 cycles at 1.0 A g^−1^ with a remarkable capacity retention of 98.8% and average Coulombic efficiency as high as 99.52% for the same cycle period. The excellent electrochemical performance of Si@C composites can be attributed to the intrinsic structural characteristics along with rationally macroporous design that maintains the electrode integrity and stabilizes the electrode/electrolyte interface.

## Experimental Section

### Synthesis of SiO_2_ From Fresh BLs

Fresh BLs were collected from Anji, one of the most important producing places of bamboos in China. Dried BLs were obtained by washing away dirt with water and drying at 80°C overnight. Subsequently, the BLs were further leached in a 1 M HCl solution for 10 h to remove metal impurities and then washed with deionized water. After drying overnight, high-purity SiO_2_ with a BL-like appearance was produced by calcining in air at 700°C for 5 h to remove organic components in BLs.

### Synthesis of **3D** **Macroporous Si**

SiO_2_ microparticles were obtained by milling the BL-derived SiO_2_ for 2 h at 200 rpm (Pulverisette Premium Line 7, Fritsch, Germany). Afterwards, the SiO_2_ microparticles, Mg powder and NaCl were mixed uniformly in a mortar based on a molar ratio of 1:1.2:4.92. The mixture was transferred to a corundum boat, which was then placed in a tube furnace and heated to 700°C at a ramp rate of 5°C min^−1^. After heating for 2 h, the obtained powder was immersed in a 1 M HCl solution and hydrofluoric acid (10 wt%) successively to remove MgO and residual SiO_2_, respectively.

### Synthesis of 3D Macroporous Si@C Composites

The 3D macroporous Si@C composites were obtained via a simple carbon coating procedure. 3D macroporous Si and polyvinylidene fluoride (PVdF) were dispersed in N-Methyl pyrrolidone (NMP) for 24 h at a mass ratio of m_Si_: m_PVdF_ = 1:1. After drying in vacuum, the precursor mixture was transferred into a tube furnace and calcinated at 550°C for 3 h with a ramp rate of 5°C min^−1^ under Ar atmosphere. Finally, the dark powder of 3D macroporous Si@C composites was obtained.

### Characterization of Materials

Morphological observations of samples were characterized using a field emission scanning electron microscope (FESEM, Hitachi S-4700) and a transmission electron microscope (TEM, Tecnai G2 F30, FEI). Elemental analyses were performed on an energy dispersive spectroscopy (EDS) attached to the SEM apparatus. Structural information was obtained using an X-ray diffractometer (XRD, PANalytical X’Pert Pro). Raman spectroscopy was recorded with an Edinburgh RM5 Raman microscope. Thermogravimetric (TG) analysis was conducted by thermogravimetric analyzer (STA 449 F3). The Brunauer-Emmett-Teller (BET) surface areas and the Barrett-Joyner-Halenda (BJH) pore distribution of the samples were measured by the Nitrogen adsorption−desorption method (ASAP 2000, Micromeritics).

### Electrochemical Measurements

To prepare the working electrodes, active materials, sodium carboxylmethyl cellulose, and carbon black were mixed uniformly in deionized water at a mass ratio of 7:1.5:1.5 to form a slurry. The slurry was then cast onto a copper foil, followed by drying at 60°C for 10 h in vacuum. The working electrodes were punched into circular discs with loading of Si active materials typically around ∼1.0 mg cm^−2^. The CR2025-type cells were assembled in an Ar-filled glovebox, in which oxygen and H_2_O content are less than 0.1 ppm. Li metal foils were used as the counter electrodes, while 1 M LiPF_6_ dissolved in a mixed solution of ethylene carbonate/diethyl carbonate (EC/DEC = 1:1, v/v) with 3 vol% vinylene carbonate was used as the electrolyte. For battery test, the cyclic voltammetry (CV) measurements were carried out by using a CHI420C electrochemical quartz crystal microbalance at a scanning rate of 0.1 mV s^−1^. The charging and discharging measurements of the batteries were conducted using a Land CT 2001A system in a voltage range of 0.01–1.0 V.

## Results and Discussion

**SCHEME 1:**
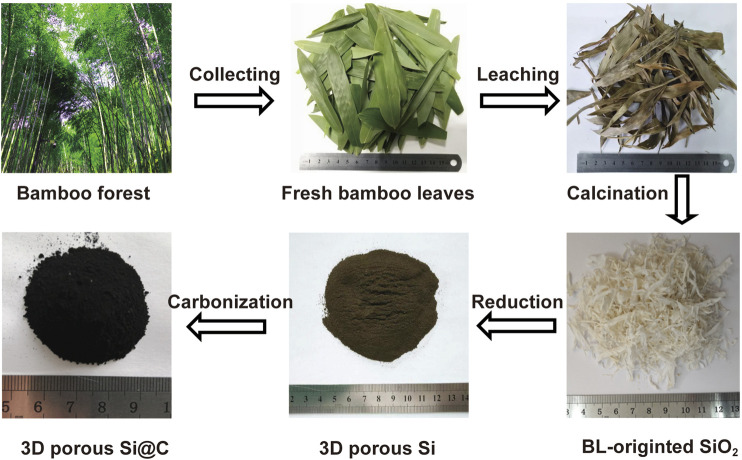
Flow chart for the synthetic procedures of the 3D macroporous Si@C composites derived from BLs.

Scheme 1 illustrates the flow chart for the synthesis process of 3D macroporous Si@C composites using BLs as the resource. The collected BLs were first cleaned with water to remove dirt, following by hydrochloric acid pickling to remove metal impurities. After leaching, the color of the fresh BLs turned from verdant to brown. The organic components were removed by calcining the dried BLs under air, resulting in white SiO_2_ flakes. Compared with leached BLs, the shrinkage in the size of the SiO_2_ precursor can be observed, while the overall appearance of the BL-like SiO_2_ remains intact. The SiO_2_ precursor was transformed into brown Si powder via magnesiothermic reduction after subsequent removal of MgO byproduct and residual SiO_2_. Finally, 3D macroporous Si@C composites was obtained as dark powder after carbon coating.

**FIGURE 1:**
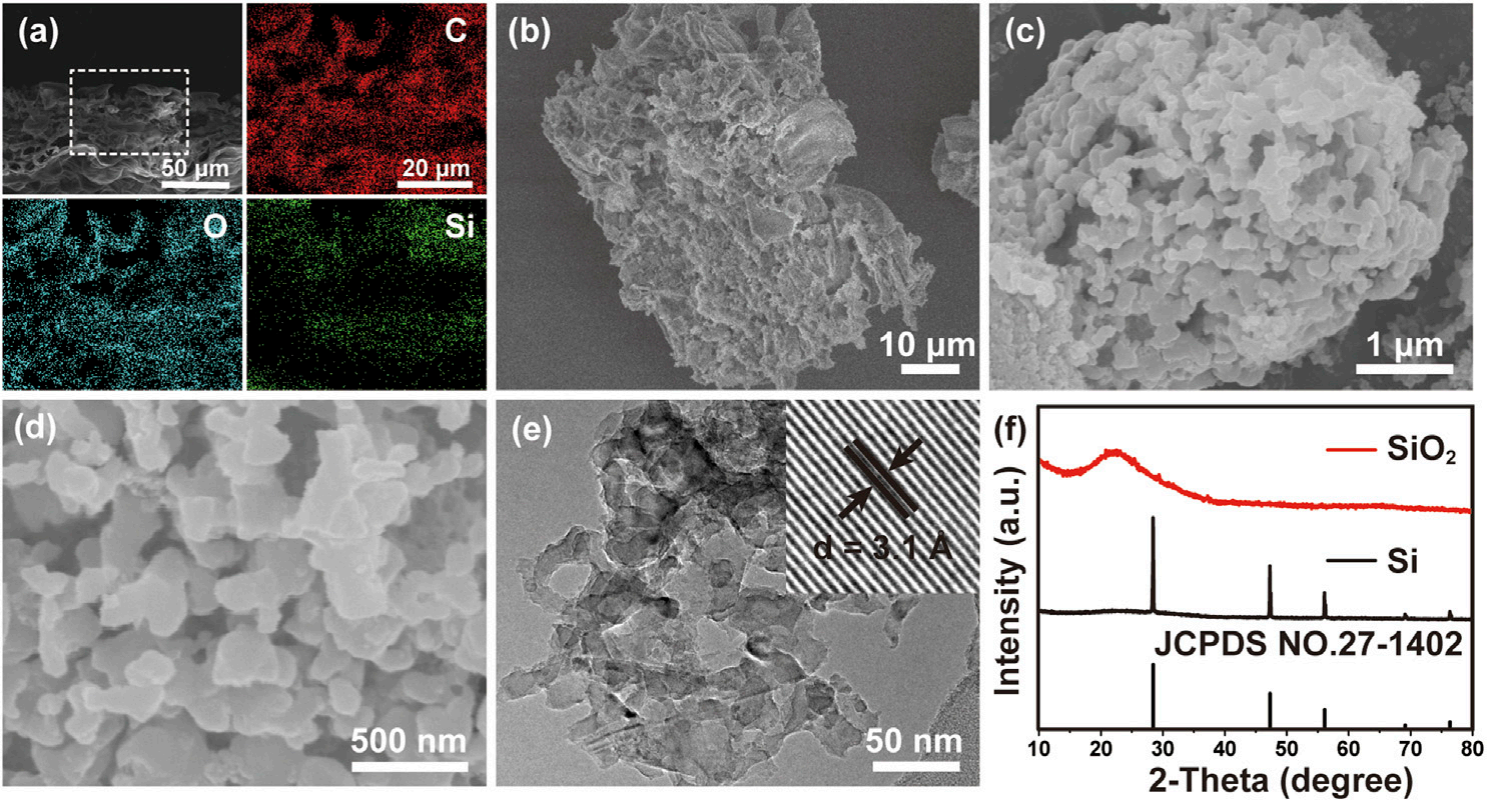
**(A)** EDS mappings of a fresh BL with a cross-sectional view. SEM images of **(B)** SiO_2_ precursor and **(C,D)** 3D macroporous Si. **(E)** TEM and HRTEM (inset) images of porous silicon. **(F)** XRD patterns of SiO_2_ precursor and porous silicon.

Figure 1A shows the EDS mappings of a fresh BL with a cross-sectional view, in which C, O and Si are uniformly distributed from top to bottom. A large number of pores can be observed in fresh BL, indicating its natural porous structure. An elemental analysis confirms that the fresh BLs mainly consist of C, O and Si (Supplementary Figure S1). The SiO_2_ content in the leached BLs was evaluated to be 21.43% by TG as shown in Supplementary Figure S2. After thermally decomposing the organic matter, the SiO_2_ skeleton revealed an interconnected porous network as presented in Figure 1B with an atomic ratio of Si:O near 1:2 (Supplementary Figure S3A). To reduce the particle size of the SiO_2_ precursor, a simple ball milling process was applied to the SiO_2_ to produce SiO_2_ microparticles (Supplementary Figure S4). Using the SiO_2_ microparticles as source, porous Si can be obtained via a magnesiothermic reduction method. Figures 1C,D show 3D porous Si with an interconnected network, implying the successful structural preservation of SiO_2_. This unique structure is affirmed by the TEM image (Figure 1E), while the high-resolution TEM (HRTEM) image (Figure 1E, inset) shows the lattice frings corresponding to Si 111) planes (Liu et al., 2015). The successful reduction of SiO_2_ to Si is verified by EDS (Supplementary Figure S3B), in which a distinct decrease of O content in the sample can be noticed compared with that of the SiO_2_ precursor. In addition, the XRD patterns (Figure 1F) of the samples before and after magnesiothermic reduction further confirms the conversion of amorphous SiO_2_ precursor to crystalline Si.

The structural evolution can also be identified from Nitrogen adsorption isotherms in Supplementary Figure S5A. The specific surface area of the samples decreases from 204 m^2^ g^−1^ for original SiO_2_ precursor to 130 m^2^ g^−1^ for the ball-milled SiO_2_, indicating the ball milling process disrupt the porous structure to a certain extent. The magnesiothermic reduction reaction further reduces the specific surface area of Si to 86 m^2^ g^−1^, which can be ascribed to the mergence of the pristine pores from the BLs with newly formed pores during the removal of MgO by-product and unreacted SiO_2_ by acid etching treatment. The pore size distribution curves (Supplementary Figure S5B) show that the sharp peaks of original and ball-milled SiO_2_ are both located at around 35 nm, indicating the ball milling does not change the pore size of SiO_2_. However, the pore size distribution curve of porous Si (Supplementary Figure S5C) displays a higher proportion of macropores. This implies that magnesiothermic reduction reaction and subsequent acid etching introduce more empty space into the structure, leading to the expansion of the pristine pores. It should be mentioned that macropores are beneficial to improve cycling stability of the anode materials compared to nanopores and mesopores, since they can better buffer the huge volume change during lithium insertion and facilitate the diffusion of ions (Li et al., 2014).

**FIGURE 2:**
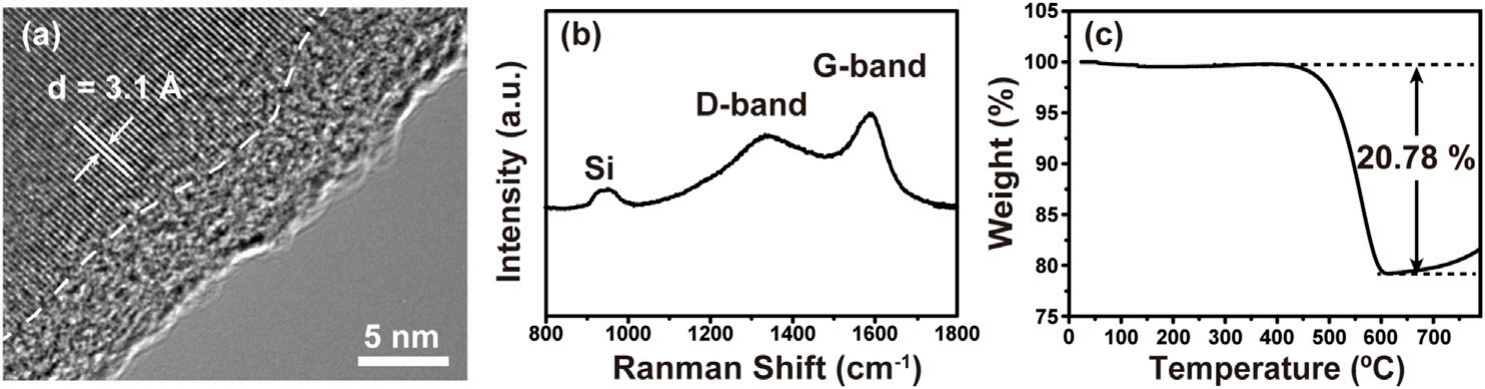
**(A)** HRTEM image of macroporous Si@C composites. **(B)** Raman spectrum of macroporous Si@C composites. **(C)** TGA curve of macroporous Si@C composites.

Figure 2A shows the HRTEM image of porous Si@C composite, in which the interface between amorphous carbon layer and crystalline silicon can be clearly observed. The thickness of the carbon layer is around 6 nm, while the characteristic lattice spacing of 0.31 nm corresponds to Si 111) planes. The Raman spectrum of the macroporous Si@C composites in Figure 2B shows a small peak at about 950 cm^−1^, indicating the typical vibration modes of the crystalline Si. A broad peak centered at 1,320 cm^−1^ and a sharp peak at 1,600 cm^−1^ correspond to the D band and the G band of carbon, respectively, confirming its amorphous nature (Jung et al., 2018; Zhu et al., 2019). As shown in Figure 2C, the carbon content in macroporous Si@C composites is measured to be 20.78%.

**FIGURE 3:**
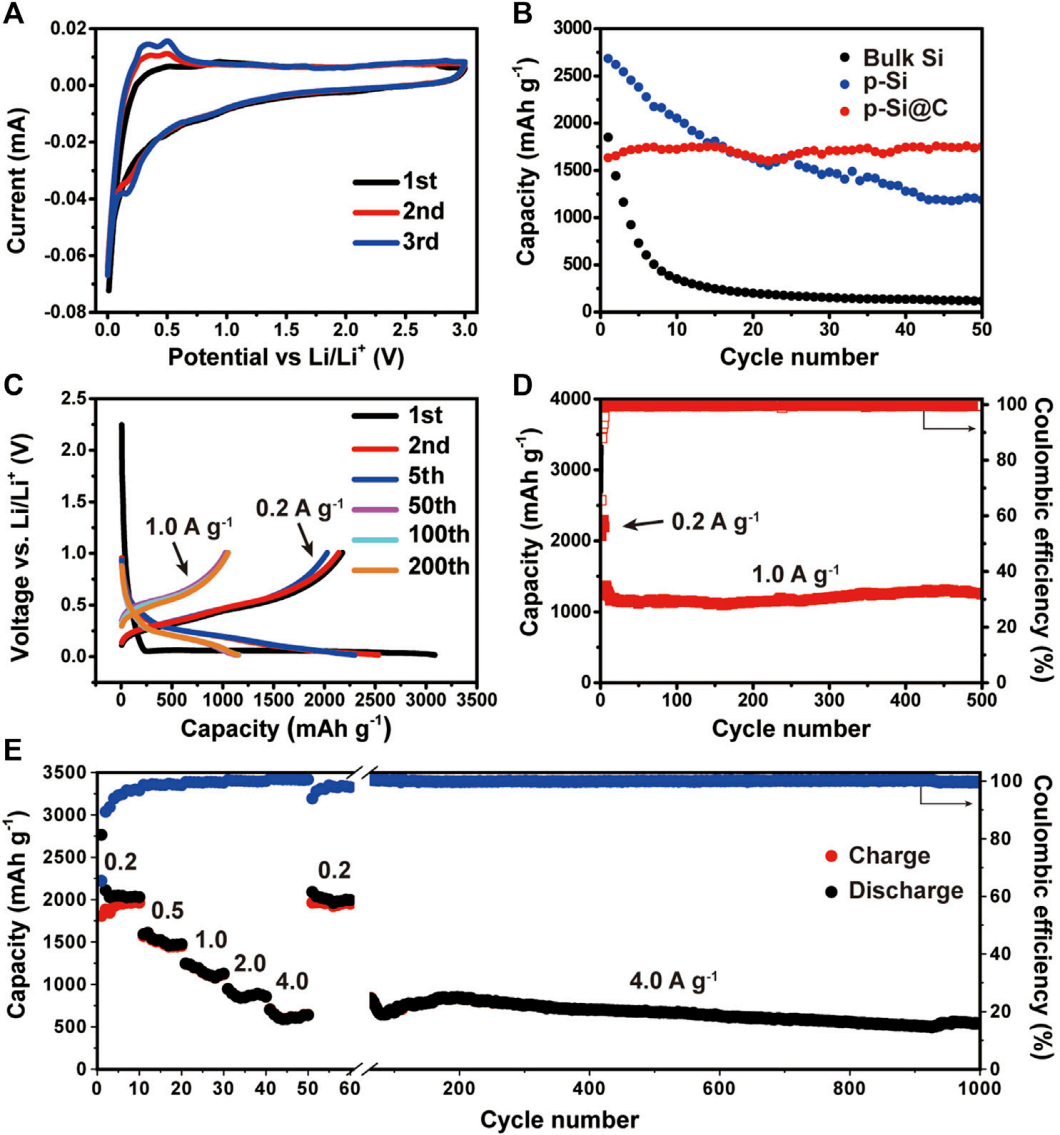
**(A)** CV curves of macroporous Si@C composites. **(B)** Cycling performance of bulk silicon, macroporous silicon, and macroporous Si@C composites at the same current density of 0.5 A g^−1^. **(C)** Voltage profiles of the macroporous Si@C composites for the first, second and fifth cycles at 0.2 A g^−1^ and the 100th, 200th and 500th cycles at 1.0 A g^−1^. **(D)** Cycling performance of macroporous Si@C composites. **(E)** Rate capabilities of macroporous Si@C composites under different current densities.

The lithium storage performance of 3D macroporous Si@C composites were evaluated by coin cells. Figure 3A shows the CV curves of the macroporous Si@C anodes for the initial three cycles. For the first discharge process, the sharp peak at 0.01 V can be ascribed to the alloying reaction of the crystalline silicon transforming to amorphous Li_x_Si. For the first charge process, two distinct peaks at 0.34 and 0.51 V correspond to the phase transition from Li_x_Si to amorphous silicon (Limthongkul et al., 2003; Obrovac and Krause, 2007). The gradual increase of the peak intensity in the discharge process indicates the activation or partial reconstruction of silicon during the initial cycles, which allows more active materials to participate in the lithiation reaction during subsequent cycles.

To evaluate the cycling performance, the charge/discharge galvanostatic cycling of the 3D macroporous Si@C composites, porous Si and bulk Si were tested at the same current density of 0.5 A g^−1^. As shown in Figure 3B, the macroporous Si@C composites exhibit the lowest initial reversible capacity of 1,633.6 mAh g^−1^ compared with the other two anodes. This result can be expected because the coating of carbon would inevitably reduce the reversible capacity. Nonetheless, the macroporous Si@C composites delivers the highest reversible capacity with a capacity retention of 100% after 50 cycles. In contrast, bulk Si exhibit rapid capacity decay during the 50 cycles with a capacity retention of only 6.4%, while the bare porous Si deliver a capacity retention of only 44.2%. The outstanding cycling stability of the macroporous Si@C composites can be attributed to the durability of the natural silicon framework inherited from BLs and the macroporous structure that accommodates the volume expansion of silicon during repeated Li insertion/extraction. Moreover, the amorphous carbon layer on the surface macroporous Si@C composites can further maintain the integrity of the electrodes throughout the cycling. The voltage profiles of the macroporous Si@C composites are presented in Figure 3C. The plateaus shown in the curves are in good agreement with the peaks appeared in CV curves. The charge curves of these cycles mostly overlap, indicating high reversibility of the porous Si@C electrodes.

Prolonged cycling test was conducted on the macroporous Si@C anodes to further evaluate the cycling performance. As shown in Figure 3D, the macroporous Si@C anodes exhibit superior stability and deliver a reversible capacity of the 1,247.7 mAh g^−1^ after 500 cycles at the current density of 1.0 A g^−1^. The capacity retention reaches 98.8%, corresponding to a 0.0024% capacity decay per cycle. Despite the relatively low initial Coulombic efficiency (65.6%), the subsequent Coulombic efficiencies of the macroporous Si@C composites rise rapidly to above 99% after the initial five cycles. The average Coulombic efficiency of the following cycles is 99.52% at the current density of 1.0 A g^−1^. The remarkable cycling stability and Coulombic efficiency of the macroporous Si@C materials can be attributed to the robust and unique porous structure of the anodes that accommodates the large strain during cycling. Apart from this, the surface carbon layer also stabilizes the structure in preventing repeated fracture and the growth of SEI layer on the surface of the anodes.

The rate capabilities of the macroporous Si@C anodes are shown in Figure 3E. The macroporous Si@C anodes exhibit reversible capacities of 1963.6 and 641.7 mAh g^−1^ at the current density of 0.2 and 4.0 A g^−1^, respectively. When the current density returns to 0.2 A g^−1^, 99.4% of the reversible capacity can be recovered. The macroporous Si@C composites still reach a reversible capacity of 538.2 mAh g^−1^ after subsequent almost 1,000 cycles at a high current density of 4.0 A g^−1^, corresponding to a capacity retention of 83.9%. The excellent rate capabilities of the porous Si@C composites can be owing to the 3D interconnected porous structure, which facilitates the infiltration of the electrolyte into the empty space inside the network and shortens the diffusion distance of lithium ions. In addition, the carbon coating further enhances the electronic conductivity of the anode materials and benefits the electron transportation at high rates. Table 1 summarizes the electrochemical performance of Si-based anodes fabricated via magnesiothermic reduction reported in previous works. In comparison with other materials, macroporous Si@C composites in this work present superior lithium storage properties with respect to specific capacity, cycling stability and Coulombic efficiency.

**TABLE1:** Comparison of electrochemical performance with other Si-based anodes fabricated via magnesiothermic reduction.

Raw Material	Anode Material	Current Density (A g^−1^)	Cycles	Reversible Capacity (mAh g^−1^)	Coulombic Efficiency	Capacity Retention (%)	References
Rice husks	3D nano-Si	6.0	300	1,274.3	99.7% from 201th to 500th cycles on average	82	Jung et al. (2013)
Bamboo charcoal	Porous Si@N/C	0.2	120	603	>99% after the 1st cycle	37.9	Zhang et al. (2018)
Reed plants	3D porous Si@C	2.1	200	1,050	—	—	Liu et al. (2015)
Diatomite	Si/SiO_2_@C	1.0	500	877	>99.8% after 10 cycles	81.6	Wu et al. (2020)
Sea sand	Porous Si@C	0.4	200	1,000	—	—	Ahn et al. (2017)
Waste glass	Mesoporous Si	0.5	360	1,000	>98% after 5 cycles	37.9	Mu et al. (2019)
Bamboo leaves	3D macroporous Si@C	1.0	500	1,247.7	99.52% from 6th to 500th cycles on average	98.8	this work

**FIGURE 4:**
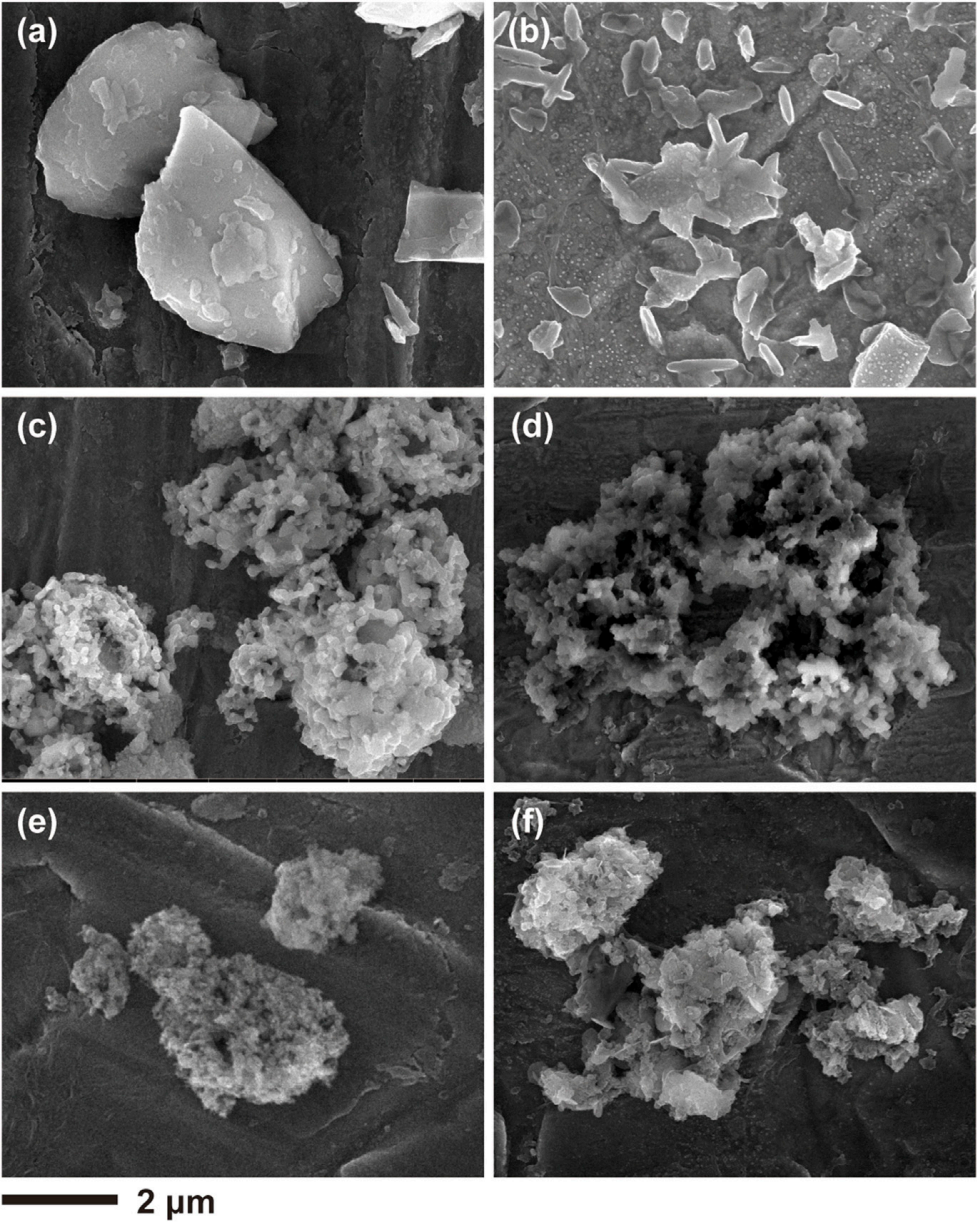
SEM images of **(A,B)** bulk silicon **(C,D)** macroporous silicon and **(E,F)** macroporous Si@C composites before and after 100 cycles.

Figure 4 shows the SEM images of the porous Si@C, porous Si and the bulk Si before and after 100 cycles. The morphological change is apparent for bulk Si, as the pristine blocks pulverize into small fragments as shown in Figures 4A,B. For the bare porous Si, the morphology of the porous particles can still be identified despite a certain degree of pulverization is evident. In contrast, the porous Si@C particles remains integrated after 100 cycles, implying that the robustness of the porous interconnected network and the carbon coating are critical to maintain electrode integrity and enhance cycling stability.

## Conclusion

In conclusion, we have demonstrated a scalable, simple and low-cost route to produce 3D macroporous Si using BLs as a sustainable and abundant resource. The resultant Si preserves the interconnected network from the natural structure of BLs and incorporates newly formed macropores. Further carbon coating is applied to the porous Si to enhance its structural durability. When used as an anode material for LIBs, the 3D macroporous Si@C composites exhibit superior cycling stability and outstanding rate capabilities. The excellent electrochemical performance can be attributed to the effective buffer of the macroporous structure, robustness of the natural network and constraint of surface carbon coating. The BL-derived 3D macroporous Si@C composites have been demonstrated as a promising anode material for next-generation high-performance LIBs.

## Data Availability

The original contributions presented in the study are included in the article/Supplementary Material, further inquiries can be directed to the corresponding authors.
