# Supplementary material for: Engineering Bamboo Leaves Into 3D Macroporous Si@C Composites for Stable Lithium-Ion Battery Anodes
Source: Front Chem. 2022 Apr 7;10:882681. doi: 10.3389/fchem.2022.882681 (PMC9021544; doi:10.3389/fchem.2022.882681)
Supplement: Supplementary file 1 [file datasheet1.pdf]

Supplementary Material

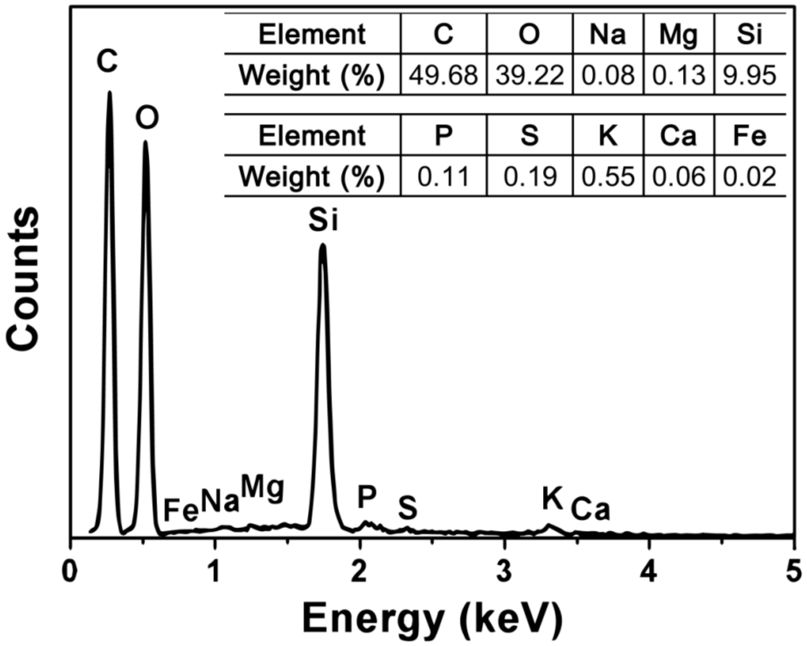

Figure S1. EDS of fresh bamboo leaves.

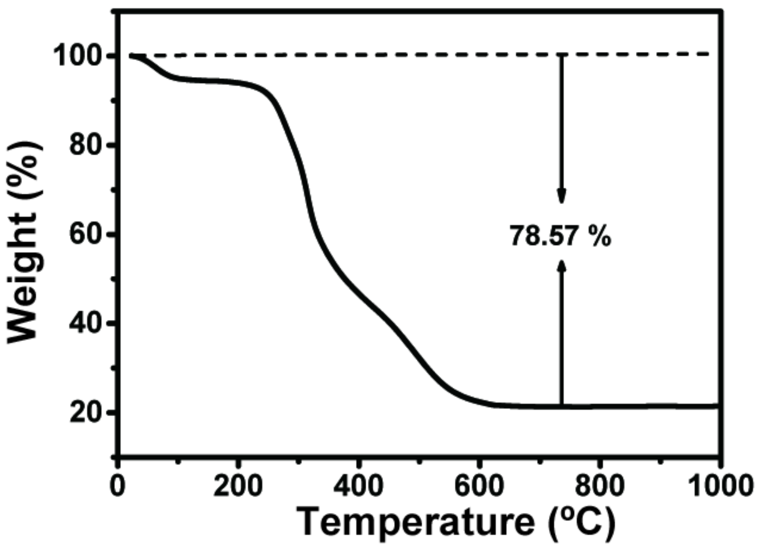

Figure S2. TG curve of HCl-leached bamboo leaves.

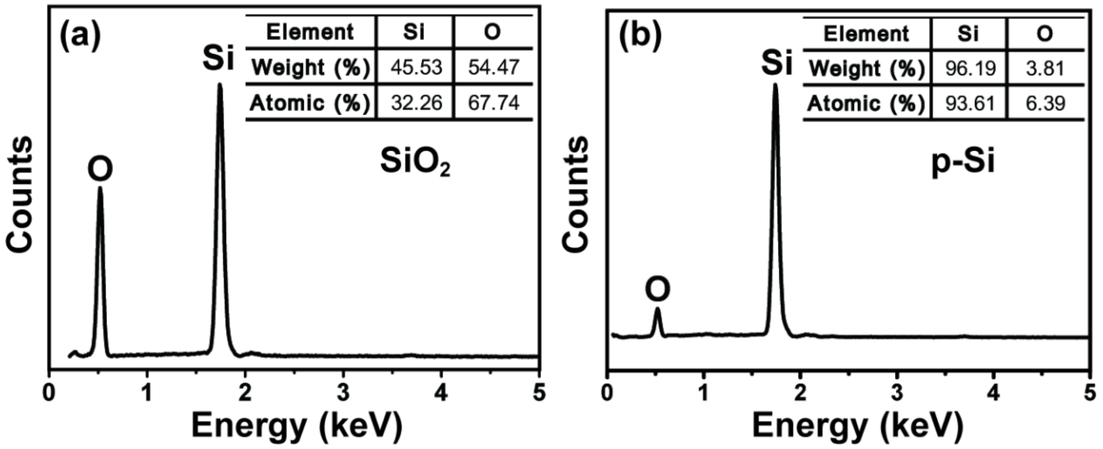

Figure S3. EDS of (a) SiO_2 precursor and (b) porous silicon.

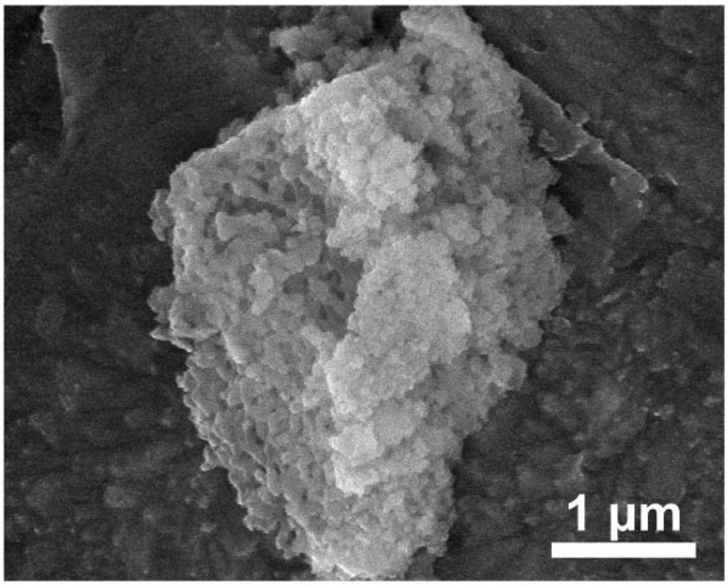

Figure S4. SEM image of a SiO_2 microparticle after ball milling.

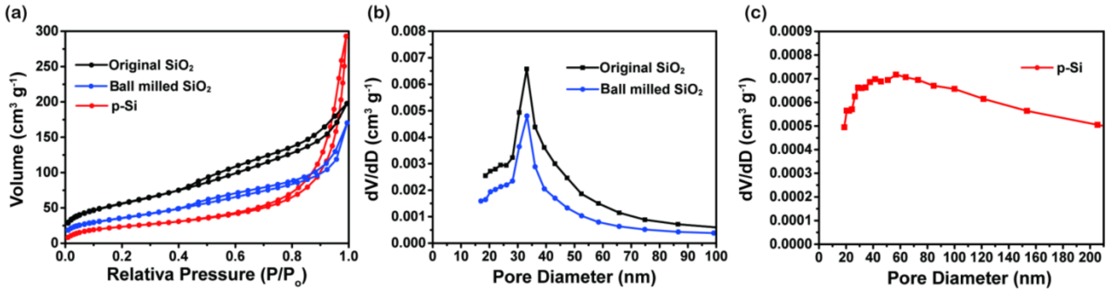

Figure S5. (a) Adsorption-desorption isotherms of original SiO_2, ball-milled SiO_2 and porous silicon. (b) The pore-size distributions of original SiO_2 and ball-milled SiO_2. (c) The pore-size distribution of porous silicon.
